# Cattell-Braasch Maneuver: A Gadget to Manipulate Abdominal Aortic Aneurysm in a Patient with a Left-Sided Inferior Vena Cava

**DOI:** 10.1155/2019/9789670

**Published:** 2019-12-20

**Authors:** Akiko Tobe, Takuro Shirasu, Takatoshi Furuya, Motoki Nagai, Yukihiro Nomura

**Affiliations:** Department of Surgery, Asahi General Hospital, Chiba, Japan

## Abstract

A 76-year-old man was diagnosed with abdominal aortic aneurysm and a left-sided inferior vena cava. He underwent open surgery, and we employed the Cattell-Braasch maneuver to approach the abdominal aortic aneurysm from the right side. This enabled securing of the abdominal aortic aneurysm neck without mobilizing or dissecting the inferior vena cava. His postoperative course was uneventful. Although abdominal aortic aneurysm is typically approached from the left side in open surgery, approaching from the right side is beneficial in patients with abdominal aortic aneurysm and a left-sided inferior vena cava.

## 1. Introduction

The presence of a left-sided inferior vena cava (LIVC) poses a challenge during open surgery when present with abdominal aortic aneurysm (AAA). The LIVC usually obscures the aortic neck, and proximal anastomosis becomes much more difficult. However, surgical approaches to treat AAA with LIVC have not been well discussed in the literature, partly because this anomaly is rare, with a reported incidence of 0.1%–0.4% [1]. We report the case of a 76-year-old man, whom we successfully treated for AAA with LIVC by approaching the AAA from the right side in open surgery.

## 2. Case Presentation

**Figure 1:**
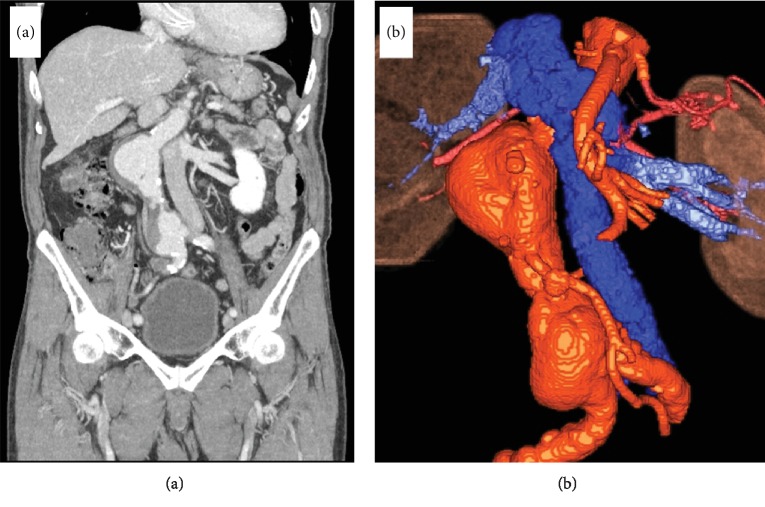
Infrarenal abdominal aortic aneurysm and left-sided inferior vena cava. (a) Computed tomography revealed that the inferior vena cava ran left to the abdominal aortic aneurysm (AAA). The AAA was 50 mm in diameter. (b) The left-sided inferior vena cava crossed the aortic neck anteriorly after receiving the left renal vein at the level of the bilateral renal arteries and ran right to the aorta in the normal position.

A 76-year-old man, who was being monitored for thoracic aortic aneurysm using computed tomography, was first diagnosed with a small AAA 6 years before undergoing surgery. Simultaneously, he was diagnosed with LIVC. Both of his common iliac veins flowed into the infrarenal IVC, which then ran parallel to the left side of the aorta and AAA. The LIVC crossed the aortic neck anteriorly after receiving the left renal vein at the level of the bilateral renal arteries and ran right to the aorta in the normal position. The AAA was located immediately dorsal to the head of the pancreas and duodenum. The left gonadal and adrenal veins were direct tributaries of the LIVC (Figure 1). His AAA rapidly increased by 10 mm in 1 year and finally enlarged to a diameter of 50 mm. After fully receiving an explanation of his condition, he opted to undergo open surgery for the AAA.

The patient's medical history included chronic kidney disease, hypertension, and a complete atrioventricular block, which had been treated with a cardiac pacemaker implantation. The cortex of the right kidney had already decreased in size since AAA diagnosis. The serum creatinine level was 1.26–1.31 mg/dL throughout the 6-year follow-up period. He had been smoking approximately 40 cigarettes per day for 50 years and had quit smoking 8 years before the surgery.

**Figure 2:**
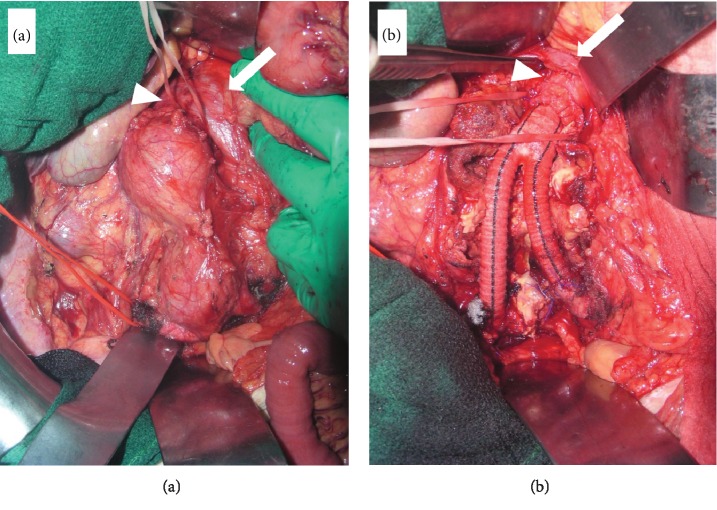
Surgical view of the abdominal aortic aneurysm and left-sided inferior vena cava. (a) After mobilizing the duodenum, right colon, and intestine by employing the Cattell-Braasch maneuver, the aorta was clearly observed from the neck of the abdominal aortic aneurysm (AAA) to both of the common iliac arteries. (b) A bifurcated artificial graft was implanted from the infrarenal aorta to the bilateral common iliac arteries. Arrows indicate the left-sided inferior vena cava. Arrowheads indicate the neck of the AAA.

For the surgery, we employed a transperitoneal approach with right-sided medial visceral rotation. We mobilized the duodenum, the head of the pancreas, and the right-sided colon, which provided a clear view of the AAA and LIVC without dividing any major blood vessels. Without division or mobilization of the LIVC, the infrarenal aortic neck was dissected. The bilateral common iliac arteries were also dissected for clamping in the surgical field (Figure 2). A bifurcated artificial graft (Hemashield Gold 14 × 8, MAQUET Holding B.V. & Co. KG, Germany) was implanted from the infrarenal aorta to the bilateral common iliac arteries (Figure 2). The duration of the operation was 2.9 h, and the estimated blood loss was 210 mL. The postoperative course was uneventful. The patient recovered his normal physical status and was discharged on day 7 after surgery.

## 3. Discussion

We rarely encounter LIVC in patients with AAA. Since first reported by Davachi et al. in 1965 [2], some reports have been published in the literature to date. Almost all published reports described open surgery, which can be challenging, particularly when securing the AAA neck. In cases with LIVC, the AAA necks are supposed to be obscured by the IVC. According to normal IVC embryology, the right renal vein's predecessor persists and the IVC runs right to the aorta. In patients with LIVC, the left renal vein's predecessor persists, the right renal vein's predecessor atrophies during the embryonal period during weeks 6-8, and the IVC runs left of the aorta [3]. Consequently, the IVC crosses the aorta anteriorly from the left to the right side at the level of the renal arteries, and the part of the IVC that crosses to the aorta corresponds to the left renal vein in normal embryology. Therefore, the infrarenal AAA neck is usually located immediately dorsal to the point of crossing of the IVC. This specific anatomy can complicate open surgery.

We will discuss the surgical approach to the aneurysm coinciding with this uncommon variant of IVC. We employed right-sided medial visceral rotation or the Cattell-Braasch maneuver to approach the AAA neck. This maneuver is beneficial for exposing the IVC in cases of normal anatomy. In patients with LIVC, this approach can be modified to dissect the infrarenal aorta without dividing the LIVC. Conversely, if we approach the AAA neck from the left of the Treitz ligament, as is often performed in cases of normal anatomy, the root of the mesentery and the crossing point of the LIVC would become obstacles to dissecting the AAA neck. We searched the literature for studies pertaining to AAA with LIVC, which described (or the author directly answered) the approach to the aneurysm; there was no restriction with regard to the date of publication (Table 1) [4–12]. We reviewed 9 case reports describing 10 open surgeries. We used the phrase right-sided open surgery approach, when the AAA and AAA neck were approached by mobilizing the right-sided visceral organs. In the left-sided approach, the AAA and AAA neck were approached from the left of the Treitz ligament. Left-sided approach frequently involved mobilization of the IVC by resection of some tributaries or division and reanastomosis of the IVC. All nine patients who were treated using the left-sided approach required IVC treatment as mentioned earlier, whereas those treated using the right-sided approach required no further treatment for IVC. Division and reconstruction of the IVC should be avoided if possible because dividing the IVC or its tributaries poses a high risk of bleeding. However, the Cattell-Braasch maneuver involves the risk of injury to the duodenum, pancreas and ascending colon, and right ureter and paralytic ileus as described previously [13]. Another disadvantage of adopting the Cattell-Braasch maneuver would be poor access to the suprarenal part of the abdominal aorta. When combined with the retroperitoneal incision from the aortic bifurcation along the inferior mesenteric vein to the ligament of Treitz, the Cattle-Braasch maneuver can provide a clear surgical field for suprarenal regions [14]. However, even this procedure does not allow access to the paravisceral region of the abdominal aorta, which can be better manipulated in the retroperitoneal approach.

In our patient as well as in cases reported previously, AAAs were located in the infrarenal region of the abdominal aorta. Interestingly, some cases including ours described the formation of the AAA at the inflection point of the angulated aortic axis. We need to scrutinize more cases to determine the possible reason for this, such as hemodynamic influences, but the Cattell-Braasch maneuver is more appropriate in such cases, since those will have infrarenal normal neck for proximal control. Endovascular aortic repair (EVAR) may also be suggested for this complex anatomy. In fact, we had proposed EVAR as an option to the patient, but he chose open surgery to minimize the necessity for reinterventions in the long term.

The difference between the transperitoneal and retroperitoneal approaches in patients with LIVC has been reported previously [11, 12]. According to Dimic et al., retroperitoneal approach would complicate the aortoiliac procedure [12]. In patients with normal anatomy, the retroperitoneal approach is generally useful for more proximal aneurysms like pararenal and suprarenal AAA, and there is no significant difference in terms of the rates of perioperative morbidity and mortality between transperitoneal and retroperitoneal approach for AAA repair [15]. As mentioned earlier, proximal anastomosis is key in the management of this anomaly, for which the retroperitoneal approach might be helpful. However, patients with AAA sometimes have iliac artery aneurysms, which require simultaneous treatment. Although the decision must be made on a case-by-case basis, the transperitoneal approach is more suitable than the retroperitoneal approach in cases of LIVC because it provides a larger surgical field caudally.

In conclusion, right-sided medial visceral rotation or the Cattell-Braasch maneuver was useful in open surgery for AAA with LIVC.

## Figures and Tables

**Table 1:** Previous reports of abdominal aortic aneurysm with left-sided inferior vena cava.

	Author (publication year)	Age (years)	Sex	Incision	Approach	Techniques for IVC	Complications	Outcome	AAA type	Crossing point
1	Perler (1989)	64	M	R	Left	Mobilization	None	Alive	I	On the aortic neck
2	Gargiulo (1994)	57	M	T	Left	Division and reconstruction	None	Alive	I	On the aortic neck
3	Ishibashi (1997)	63	M	T	Left	Mobilization	None	Alive	I	Left renal vein
4	Tsukamoto (2000)	71	M	T	Left	Mobilization	None	Alive	I	On the aortic neck
5	Nishimoto (2002)	78	M	R	Right	None	None	Alive	I	Renal veins
6	Nishibe (2004)	70	M	T	Left	Mobilization	None	Alive	I	On the aortic neck
7	Radermecker (2008)	64	F	T	Left	Mobilization	None	Alive	I	On the aortic neck
8	Niino (2012)	82	M	T	Left	Mobilization	None	Alive	I	On the aortic neck
9	Dimic (2016)	68	M	T	Left	Mobilization	None	Alive	I	On the aortic neck
10	Dimic (2016)	60	M	T	Left	Division and reconstruction	DVT	Alive	NA	On the aortic neck
11	Tobe (2019)	78	M	T	Right	None	None	Alive	I	On the aortic neck

Abbreviations: AAA: abdominal aortic aneurysm; DVT: deep vein thrombosis; F: female; M: male; NA: not available; I: infrarenal; IVC: inferior vena cava; R: retroperitoneal; T: transperitoneal.
